# Metabolic Control of Dendritic Cell Functions: Digesting Information

**DOI:** 10.3389/fimmu.2019.00775

**Published:** 2019-04-25

**Authors:** Stefanie K. Wculek, Sofía C. Khouili, Elena Priego, Ignacio Heras-Murillo, David Sancho

**Affiliations:** Immunobiology Laboratory, Centro Nacional de Investigaciones Cardiovasculares (CNIC), Madrid, Spain

**Keywords:** dendritic cell, metabolism, mitochondria, glycolysis, mammalian target of rapamycin, hypoxia-inducible factor, AMP-activated protein kinase, DC subsets

## Abstract

Dendritic cells (DCs) control innate and adaptive immunity by patrolling tissues to gather antigens and danger signals derived from microbes and tissue. Subsequently, DCs integrate those environmental cues, orchestrate immunity or tolerance, and regulate tissue homeostasis. Recent advances in the field of immunometabolism highlight the notion that immune cells markedly alter cellular metabolic pathways during differentiation or upon activation, which has important implications on their functionality. Previous studies showed that active oxidative phosphorylation in mitochondria is associated with immature or tolerogenic DCs, while increased glycolysis upon pathogen sensing can promote immunogenic DC functions. However, new results in the last years suggest that regulation of DC metabolism in steady state, after immunogenic activation and during tolerance in different pathophysiological settings, may be more complex. Moreover, ontogenically distinct DC subsets show different functional specializations to control T cell responses. It is, thus, relevant how metabolism influences DC differentiation and plasticity, and what potential metabolic differences exist among DC subsets. Better understanding of the emerging connection between metabolic adaptions and functional DC specification will likely allow the development of therapeutic strategies to manipulate immune responses.

## Metabolic Control of Dendritic Cell Development

Natural dendritic cells (DCs) present in steady state comprise type 1 conventional DCs (cDC1s), type 2 cDCs (cDC2s), double negative (CD8/CD103– CD11b–) DCs (DN-DCs), and plasmacytoid DCs (pDCs; Table 1). Natural DCs derive from myeloid progenitors in the bone marrow and require FMS-like tyrosine kinase 3 ligand (FLT3L) to differentiate via the common DC progenitor (CDP) and DC precursors (pre-DCs). In addition, other cells that are functionally similar to DCs, such as Langerhans cells (LCs), can derive from embryonic precursors. Moreover, during inflammatory settings, DCs can develop from blood monocytes (moDCs; Table 1).

**Table 1:** Dendritic cell subsets *in vivo*.

**DC subset**	**Developmental origin**	**Presence *in vivo***	**Main functional specialization**	**Selected surface markers**	**Metabolic requirements for development *in vivo* and involved signaling factors**	**Status iNOS expression**
**cDC1s**	HSC → CDP → pre-cDC; depend on FLT3L	Lymphoid-resident, peripheral tissues, blood	Cross-presentation of exogenous antigens on MHCI. Th1 & CD8+ T cell immunity against intracellullar pathogens and tumors	M: CD11c+ MHCII+ CD8α+(resident) CD103+(migratory) CD24+ XCR1+ DNGR1/Clec9A+ CD11b-/low	Reduced upon energy restriction; higher ECAR, OCR, mitochondrial mass & Δψm than cDC2; mTOR (mTORC1 & mTORC2), TSC1, PI3Kγ, AKT, PTEN, AMPK, L-Myc, Mst1/2	No (spleen)
				H: CD11c+ HLA-DR+ BDCA-3/CD141+ XCR1+ DNGR1/Clec9A+ DEC205+ CD1c-	Mst1/2	? (Some blood DCs can express iNOS)
**cDC2s**			Direct presentation of exogenous antigen on MHCII. Immunogenic CD4+ Th and regulatory T cell activation	M: CD11c+ MHCII+ CD11b+/hi SIRP1α+ CD8α- CD103-	Reduced upon energy restriction; mTOR (mTORC1 & mTORC2), TSC1	No (spleen)
				H: CD11c+ HLA-DR+ CD1c+ SIRP1α+ CD11b+ CD141- inducible CD14+	Not reported	? (Some blood DCs can express iNOS)
**DN-DCs**		Peripheral tissues, blood, spleen	Not well defined. CD8+ and CD4+ T cell priming upon uptake of cell-associated antigen suggested	M: CD11c+ MHCII+ XCR1- CD103- CD11b- (variation between tissues)	AMPK	Not reported
				H: CD11c+ HLA-DR+ CD141- sometimes CD1c+ CD206+	Not reported	
**pDCs**	HSC → CDP; depend on FLT3L	Lymphoid-resident, blood, lung (mouse), tonsil (human)	Type I interferon secretion	M: CD11c-low MHCII-lowLy6C+ B220+	mTORC1, TSC1	Not reported
				H: CD11c- HLA-DR-lowCD123+ CD303+ CD304+	mTORC1, PI3K, PKB, PTEN (*in vitro*)	No (blood)
**LCs**	Yolk-sac macro- phage, fetal liver and adult blood monocyte. Self- renew.	Epidermis and stratified epithelia, migrate to lymph node	Apoptotic cell clearance, antigen presentation to CD8+ T cells, Th17, regulatory and follicular T helper cells	M: CD11c+ MHCII+ Langerin+ CD11b+/low SIRP1α+ CD24+ EpCAM+ XCR1-	mTORC1/raptor, p14/LAMPTOR2	Yes
				H: CD11c+/low HLA-DR+ Langerin+ CD1a+ E-Cadherin+ EpCAM+	Not reported	
**moDCs**	Blood monocyte, depend on GM- CSF + M-CSF	Mainly induced upon inflammation in peripheral tissues	Context dependent: CD8+ T cell, Th1, Th2 and Th17-type immunity.	M: CD11c+ MHCII+ CD11b+ Ly6C+ CD64+ DC-SIGN+ F4/80+ CD14+ (depending on tissue)	Not reported	Tip-DCs, some i- moDCs express iNOS
				H: CD11c+ HLA-DR+ CD14+ CD141- often DC-SIGN+ CD16+ CD1c+ SIRP1α+ CD11b+	Not reported	Psoriatic Tip- DC-like cells express iNOS

*Of note, iNOS is expressed by rat DCs in the thymus but not in the spleen or pseudo-afferent lymph. Δψm, mitochondrial membrane potential; cDC1s, conventional DC type 1; cDC2s, conventional DC type 2; CDP, common DC progenitor; DN-DCs, conventional double-negative DCs; FLT3L, FMS-like tyrosine kinase 3 ligand; GM-CSF, granulocyte–macrophage colony-stimulating factor; H, human; HSC, hematopoietic stem cell; i-moDCs, inflammatory moDC-like cells; iNOS, inducible nitric oxide synthase; LCs, Langerhans cell; M, mouse; M-CSF, macrophage colony-stimulating factor; MHC, major histocompatibility complex; moDCs, monocyte-derived DCs; pDCs, plasmacytoid DCs; Th, CD4+ T helper cell; Tip-DCs; TNF/iNOS-producing-DC subset that depends on CCR2*.

### Energy Metabolism During Dendritic Cell Generation

#### Differentiation of Dendritic Cells From Monocytes With GM-CSF

**Figure 1:**
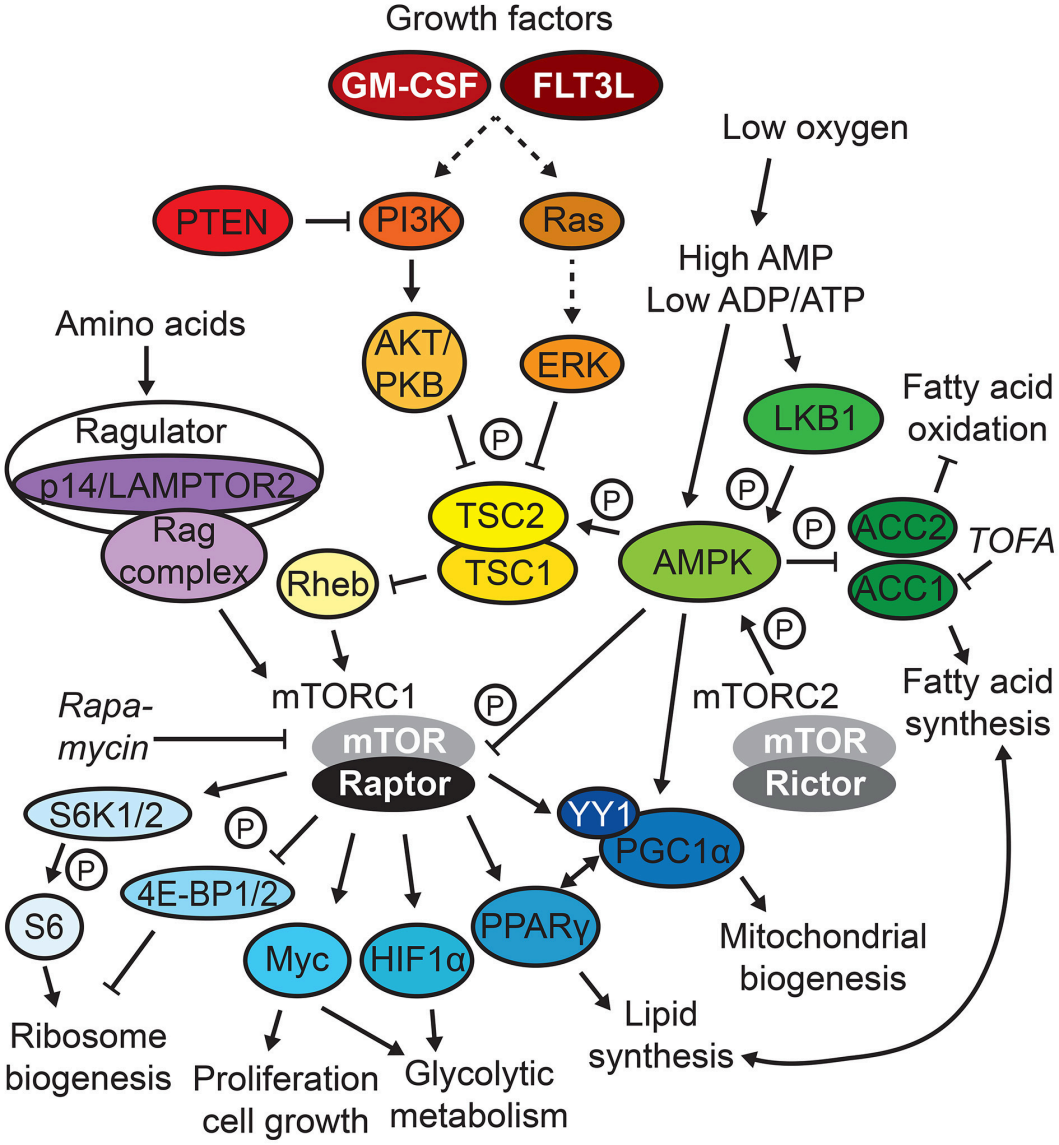
mTOR/AMPK signaling. Selected signaling circuits of the complex mammalian target of rapamycin (mTOR) and AMP-activated protein kinase (AMPK) signaling network are depicted. Frequently used metabolic inhibitors are displayed in italics, and P indicates phosphorylation.

**Figure 2:**
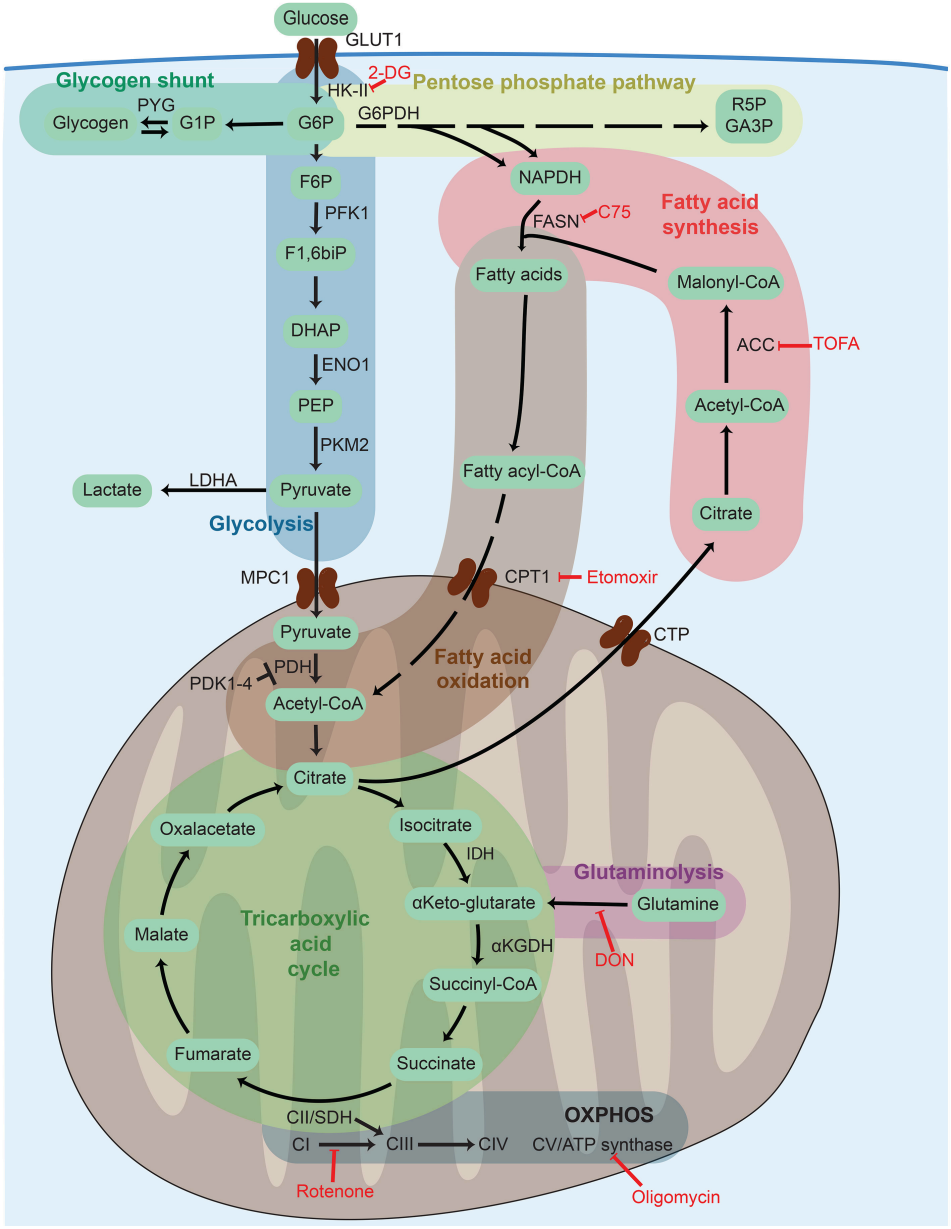
Cellular metabolism networks. Glucose is imported from the extracellular environment and can generate glycogen stores, be used in the pentose phosphate pathway to generate reducing power, or be oxidized during glycolysis to obtain adenosine triphosphate (ATP). Pyruvate generated from glycolysis can either be partially oxidized to lactate to quickly regenerate the consumed nicotinamide adenine dinucleotide (NADH) or translocate into the mitochondria to be completely oxidized thought the tricarboxylic acid (TCA) cycle. The TCA cycle can also be fueled by fatty acids via fatty acid oxidation or glutamine via glutaminolysis. The electrons released by glycolysis and the TCA cycle enter into the electron transport chain composed of complex I–V (CI–CV) where ATP is generated by oxidative phosphorylation (OXPHOS). Frequently used metabolic inhibitors are indicated in red. 2-DG, 2-deoxy-D-glucose; ACC, acetyl-CoA carboxylase; αKGDH, α-ketoglutarate dehydrogenase; CoA, coenzyme A; CPT1, carnitine palmitoyltransferase 1, CTP, citrate transport protein; DHAP, dihydroxyacetone phosphate; DON, 6-Diazo-5-oxo-L-norleucine; ENO1, enolase 1; F1,6biP, fructose 1,6 biphosphate; F5P, fructose 5 phosphate; F6P, fructose 6 phosphate; FASN, fatty acid synthase; G1P, glucose 1 phosphate, G6P, glucose 6 phosphate; G6PDH, glucose 6 phosphate dehydrogenase; GA3P, glyceraldehyde 3 phosphate, GLUT1, glucose transporter 1; HK-II, hexokinase 2; IDH, isocitrate dehydrogenase; LDHA, lactate dehydrogenase A; MPC1, mitochondrial pyruvate carrier 1; NADPH, nicotinamide adenine dinucleotide phosphate; PDH, pyruvate dehydrogenase PDK1-4, pyruvate dehydrogenase kinase 1-4, PEP, phosphoenolpyruvate; PFK1, phosphofructokinase-1; PKM2, pyruvate kinase isozyme M2; PYG, glycogen phosphorylase; R5P, ribose 5-phosphate; SDH, succinate dehydrogenase; TOFA, 5-(Tetradecyloxy)-2-furoic acid.

The importance of energy metabolism was first established in the development of human moDCs *in vitro*. Granulocyte–macrophage colony-stimulating factor (GM-CSF) and interleukin (IL)-4-induced differentiation and survival of DCs from human monocytes rely on the mammalian target of rapamycin (mTOR) complex 1 (mTORC1) activation via phosphoinositide 3-kinase (PI3K; Figure 1) and are abrogated by rapamycin, an mTOR/mTORC1 inhibitor [Table 2 and (1, 2)]. The mTORC1 downstream target peroxisomal proliferator-activated receptor γ (PPARγ) is upregulated early in moDC differentiation, affecting cell maturation and function largely through control of lipid metabolism (3–6). Indeed, inhibition of cytosolic fatty acid synthesis (FAS) via blocking acetyl-CoA carboxylase (ACC) 1 reduces moDC differentiation (7). Moreover, PPARγ co-activator-1α (PGC1α) and mitochondrial transcription factor A (TFAM), fundamental inducers of mitochondrial biogenesis and also indirect mTORC1 targets (8, 9), are also elevated during moDC differentiation (10). In line, differentiated moDCs show a higher oxygen consumption rate (OCR), contain more mitochondria, and produce more adenosine triphosphate (ATP) compared to monocytes (10, 11). Importantly, blocking the electron transport chain (ETC) with the complex I (CI) inhibitor rotenone (Figure 2) partially prevents moDC differentiation, despite causing a notable increase in glycolysis/lactate production (10, 11). Hence, moDC differentiation depends on oxidative phosphorylation (OXPHOS) and a balanced fatty acid metabolism.

**Table 2:** Culture systems of dendritic cells.

**DC culture**	**Origin**	**Culture conditions**	**Subset composition**	**Metabolic requirements of development *in vitro* and involved signaling factors**	**Status iNOS expression**
GM-DCs	Mouse bone marrow (progenitors)	GM-CSF (+IL-4), 5-7 days	DC-like and macrophage- like cells	Glucose uptake, oxygen availability, and cytosolic FAS; HIF1α	Yes (inducible)
FLT3L-DCs		FLT3L (+GM-CSF), ca. 9 days	cDC1-like cells	Glucose uptake, FAO and mitochondrial fusion/fission; higher mitochondrial mass & Δψm than cDC2; mTORC1, TSC1, PTEN, AMPK	Not reported
			cDC2-like cells	Glucose uptake & ROS; mTORC1, TSC1, PTEN	
			pDC-like cells	Glucose uptake; mTORC1, TSC1, PTEN	
iCD103-DCs		FLT3L + GM-CSF, ca. 16 days	cDC1-like cells	Not reported	No (NO measured)
moDCs	Human blood monocytes	GM-CSF + IL-4, 6-7 days	moDCs	Cytosolic FAS, mitochondrial biogenesis, active OXPHOS; mTORC1/(PI3K), PPARγ	No (depending on differentiation)

*Of note, iNOS is expressed by a mouse skin DC cell line. Δψm, mitochondrial membrane potential; FLT3L-DCs, mouse FLT3L (+GM-CSF)-induced DCs; GM-DCs, mouse GM-CSF-induced DCs; iCD103-DCs, mouse induced CD103+ DCs; iNOS, inducible nitric oxide synthase; moDCs, human GM-CSF+IL-4-induced monocyte-derived DCs*.

Likewise, the DC-like cells differentiated from mouse bone marrow cultured with GM-CSF *in vitro*, a culture system composed of a mixed population of DCs and macrophages [Table 2, GM-DCs and (12)], also show glucose uptake together with high mitochondrial membrane potential (ΔΨm) and oxygen consumption (13). Indeed, GM-DC differentiation under hypoxic conditions yields fewer total cells, and hypoxia-inducible factor (HIF)-1α deficiency further reduces the frequency of CD11c+ GM-DCs, linked to decreased ATP (14). As HIF1α is a key metabolic regulator and many of its target genes drive glycolysis (see the section Sustained Glycolysis: The Role of HIF1α), these observations point toward the importance of an active glucose metabolism involving oxidative and glycolytic pathways in GM-DCs. However, CD11c-Cre HIF1α^flox/−^ mice display unaltered DC homeostasis in the steady state (15). Moreover, impairment of cytosolic FAS by blocking ACC1 with the inhibitor 5-(tetradecyloxy)-2-furoic acid (TOFA) in GM-DC cultures or by the administration of the fatty acid synthase (FASN) inhibitor methylene-2-octyl-5-oxotetrahydrofuran-3-carboxylic acid (C75; Figure 2) *in vivo* reduces the generation of DCs (7), further suggesting that balanced FA metabolism contributes to DC development. However, it is noteworthy that the inhibitor C75 can also cause mitochondrial dysfunction (16).

#### Natural Dendritic Cell Differentiation

Generally, the presence of CDPs, pre-DCs, cDCs, and pDCs is reduced in energy-restricted mice, while myeloid progenitors, blood monocytes, and spleen macrophages are increased. FLT3L administration is unable to rescue the effect (17), highlighting the intrinsic importance of uncompromised energy metabolism for *in vivo* DC differentiation compared to monocytes. In concert, natural mouse DC progenitors in the bone marrow (Table 2; FLT3L-DC cultures) are dependent on nutrient transporters and glucose uptake for proliferation upon FLT3L stimulation *in vitro* (18). Those FLT3L-stimulated bone marrow cultures allow for the separate evaluation of mouse CDP-derived DC subsets [Table 2; FLT3L-DCs and (19)]. Notably, the inhibition of fatty acid oxidation (FAO) with etomoxir (Figure 2), promoting mitochondrial fusion with M1 or blocking fission with Mdivi-1, does not affect pDCs but strongly skews cDC differentiation toward cDC2s, while reactive oxygen species (ROS) inhibition favors cDC1s (18). Of note, apart from inhibition of carnitine palmitoyltransferase 1 (Cpt1a), a crucial enzyme for long-chain FAO, etomoxir displays off-target effects and can independently block mitochondrial respiration or enhance the ΔΨm in T cells (20). Indeed, cDC1s generally display higher mitochondrial mass and ΔΨm than cDC2s *in vitro* and *in vivo* (18, 21, 22). The non-canonical Hippo pathway kinases mammalian sterile twenty-like (Mst) 1 and 2 are crucial for mitochondrial homeostasis, energy metabolism, and immunogenic function of cDC1s, but less for cDC2s, and are activated by FLT3L in cDC1s (21). In line, *in vivo* FLT3L administration to CD11c-Cre Mst1/2^flox/flox^ mice yields reduced splenic cDC1 numbers compared to controls. Unexpectedly, CD11c-Cre Mst1/2^flox/flox^ mice exhibit elevated frequencies of splenic cDC1s, unaltered pDCs, and reduced cDC2s in the steady state (21); hence, the precise role of (non-canonical) Hippo signaling in DC development needs further investigation.

Overall, these data highlight differential energy requirements for DC subset generation, where moDCs and spleen cDC1s appear more dependent on functional mitochondrial metabolism and OXPHOS than cDC2s or pDCs (Tables 1, 2).

### Nutrient-Sensing Pathways Affecting Dendritic Cell Development

Adaption to extra- and intracellular nutrient sensing via the mTOR network composed of mTORC1 and 2 complexes (Figure 1) is central for the development of DCs (23). This notion is supported by the fact that the DC differentiation-inducing factors GM-CSF and FLT3L directly induce mTOR activation (2, 24, 25).

#### Monocyte-Derived Dendritic Cells and Embryo-Derived Langerhans Cells

The generation and survival of the non-CDP-derived human moDCs and self-maintaining LCs depend on mTORC1 (Tables 1, 2). As mentioned in the previous section, mTOR is constitutively active in cultured human moDCs, and the mTOR inhibitor rapamycin, which affects mTORC1 stronger than mTORC2, abrogates their differentiation, inducing apoptosis, in line with GM-CSF/IL-4 activating mTOR to sustain survival (1, 2). Mice deficient in the mTORC1 component Raptor in CD11c-expressing cells, but not the mTORC2 component Rictor (Figure 1), progressively lose epidermal LCs over time (26). In concert, LCs deficient in the Ragulator complex component p14 [a.k.a. lysosomal adaptor and mitogen-activated protein kinase and mTOR activator/regulator 2 (LAMPTOR2)], which display abrogated extracellular signaling-regulated kinase (ERK) and mTOR signaling, are increasingly mature and unable to self-renew due to reduced responsiveness to tumor growth factor (TGF)-β1 (27, 28), which is crucial for LC differentiation and maintenance (29).

#### Dendritic Cells Generated From Common Dendritic Cell Progenitors

Despite the Ras/PI3K/AKT/mTOR signaling axis (Figure 1) being activated by FLT3L (24, 25), the precise role of mTOR signaling is more ambiguous in FLT3L-dependent, CDP-derived DC subsets (Tables 1, 2). There are conflicting observations depending on how mTOR signaling is targeted. A line of evidence suggests that active mTOR signaling promotes generation of proper natural DC numbers and subset distribution. *In vitro*, generation of pDCs, cDC1s, and cDC2s in FLT3L-DCs is reduced by rapamycin and enhanced by loss of phosphatase and tensin homolog (PTEN), a negative regulator of PI3K/AKT/mTOR signaling (24) (Figure 1). Similarly, rapamycin administration to mice in the steady state decreases CDPs and pre-DCs in the bone marrow as well as total CD11c+ DCs, pDCs, and cDC2s in the spleen (25, 30). cDC1s and, to a lesser extent, cDC2s are profoundly reduced in the spleens and lungs of CD11c-Cre mTOR^flox/flox^ mice, CD11c-Cre Raptor^flox/flox^, Rictor^flox/flox^ double-knockout mice and mice lacking functional PI3Kγ or AKT, upstream activators of mTOR (25, 31). In accordance, cDC1s are strongly expanded in lymphoid and peripheral organs in mice deficient for PTEN (CD11c-Cre PTEN^flox/flox^ mice), a phenotype reversed by rapamycin administration (24). While pDC development is largely unaffected in PI3Kγ-deficient mice (25), human pDC differentiation *in vitro* is blocked by rapamycin, PI3K, and AKT/PKB inhibitors and facilitated by PTEN inhibition or enforced AKT activation (32).

In contrast, other reports suggest an inhibitory function of mTOR signaling for natural DC development. FLT3L-DCs show induction of AMP-activated protein kinase (AMPK) signaling, which antagonizes mTORC1 (Figure 1) (18, 33). AMPKα1 deficiency does not affect pDC or overall cDC differentiation but results in relative loss of cDC1s and DN-DCs (18, 33). Moreover, mTOR inhibition by rapamycin increases spleen cDC1 and cDC2 subsets and several DC subsets in peripheral organs upon FLT3L-mediated DC expansion *in vivo* (25). Loss of mTORC1 in DCs in CD11c-Cre Raptor^flox/flox^ mice also expands CD11c+ DCs in the bone marrow, cDC1s in the spleen, and cDC2s in the small intestine (26, 34). Similarly, tuberous sclerosis 1 (TSC1) deficiency (Figure 1), using tamoxifen-inducible Rosa-Cre TSC1^flox/flox^ mice, enhances mTOR activation and reduces pDCs, cDC1s, and cDC2s generated in FLT3L-DCs and *in vivo*, which is rescued by rapamycin (35). Conversely, CD11c-Cre TSC1^flox/flox^ mice show no major alterations in DC development (24, 36). In humans, rapamycin treatment of kidney transplant patients does not affect cDC/pDC differentiation, while DCs appear more immunogenic (2).

In conclusion, a delicate balance of the complex system of nutrient sensing and mTOR (mTORC1) signaling is crucial to ensure appropriate development of DCs (23). Strikingly, loss of both mTOR complexes results in opposite effects on *in vivo* DC development compared with loss of mTORC1 alone, probably indicating differential inhibition of mTOR downstream targets and collaboration of mTOR complexes. Indeed, DC loss upon TSC1 deficiency is accompanied by increased DC apoptosis and enhanced metabolic activity due to TSC1-dependent inhibition of Myc, an effector downstream of mTOR (Figure 1), and reversed upon Myc loss (35). Of note, Myc itself regulates glucose and glutamine catabolism in activated T cells (37). Moreover, apart from controlling mTORC1 activity, AMPK is an important regulator of fatty acid metabolism limiting ACC1/2 activity (Figure 1), which is crucial for T cell activation (38). AMPK loss generally favors cytosolic FAS over mitochondrial FAO, which likely accounts for the decrease in differentiation in AMPKα1-deficient cDC1s, as this process was shown to be sensitive to FAO block (18) and, hence, could be independent from mTOR signaling.

Moreover, the context dependence of balanced mTOR signaling in DCs may be strongly influenced by FLT3L. First, rapamycin and Mst1/2 deficiency have different or even opposing effects on DC generation in the steady state compared with FLT3L-mediated DC expansion *in vivo* (21, 25, 30). Second, while FLT3L-mediated differentiation of DC subsets from mouse bone marrow *in vitro* clearly relies on appropriate mTOR activity (18, 24, 33), GM-CSF-induced DC development *in vitro* was not affected by mTOR deregulation. FLT3L and GM-CSF have both been shown to activate mTOR (2, 24, 25); however, this activation might serve different purposes. Third, deregulated mTOR signaling appears to have stronger effects on the generation of cDC1s than other natural DC subsets, in line with spleen cDC1s being more metabolically active and their reliance on functional mitochondrial respiration (18, 21). The notion that cDC1s appear to rely more on FLT3L than other subsets, especially in peripheral tissues (39), might provide a potential explanation. Indeed, cDC1s in the spleen have higher basal phosphorylation levels of S6 protein, a readout for mTORC1 activity (Figure 1), than other DC subsets and upregulate mTOR activation to a greater extent upon FLT3L administration *in vivo*. Moreover, the increase of cDC1s upon PTEN deficiency is specific to the FLT3L-responsive CX3CR1-negative subset (24).

Last, caution has to be taken when interpreting the effect of manipulating mTOR signaling in DCs. For instance, deletion of the positive mTORC1 regulator p14/LAMPTOR2 in CD11c-expressing cells increases pre-DCs in the bone marrow and amplifies DC subsets in spleen and lymph nodes (LNs) due to accumulation of FLT3 receptor on the DC surface, leading to activation of mTOR (40). Also, while the requirement of mTOR and its signaling components was assessed, the specific mechanisms or the direct role of this nutrient sensor in regulating metabolic pathways such as glycolysis, OXPHOS, or fatty acid metabolism during DC development largely remain to be defined and could account for some of the observed controversies.

## Metabolic Rearrangements Upon Immunogenic Dendritic Cell Stimulation

**Figure 3:**
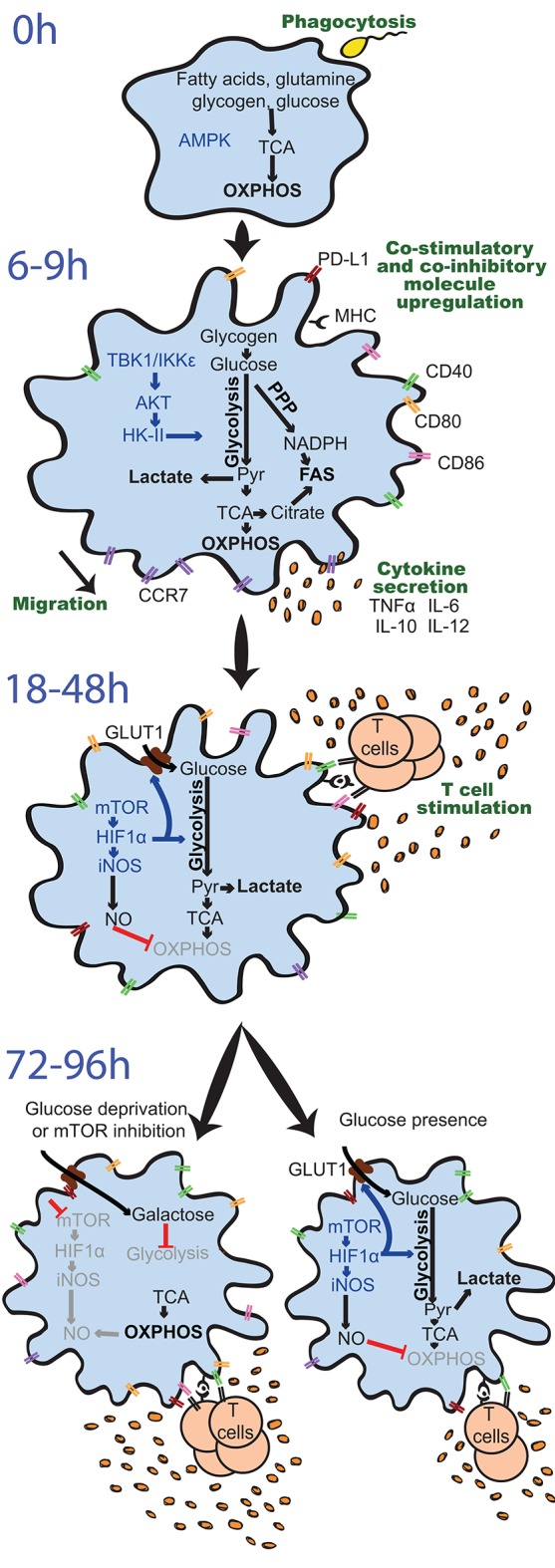
Differential regulation and effects of glycolysis induction in GM-DCs upon stimulation over time. Resting GM-DCs (top) display a basal metabolism with active AMPK (AMP-activated protein kinase) and fatty acids, glutamine, glycogen, and glucose being fully oxidized to generate energy by oxidative phosphorylation (OXPHOS). Upon early stimulation after 6–9 h, GM-DCs are activated and exhibit transiently enhanced OXPHOS/mitochondrial membrane potential and an increased glycolytic metabolism mainly using glucose from intracellular glycogen stores. The induction of glycolysis is predominantly driven by a TBK1-IKKε/AKT/HK-II axis and largely devoted to fatty acid synthesis (FAS). Moreover, enhanced early glycolytic activity of GM-DCs is vital for their migration and upregulation of co-stimulatory/inhibitory molecules as well as cytokines. At later time points about 18–48 h after robust stimulation, a mTOR/HIF1α/iNOS axis is activated in GM-DCs, leading to enforced glycolysis via upregulation of glucose importers such as GLUT1 and inhibition of OXPHOS via nitric oxide (NO). This fostered glycolytic activity appears crucial for the interaction of GM-DCs with T cells. Nevertheless, the sustained inhibition of OXPHOS by NO and reliance on glycolysis for energy generation can reduce the ability of GM-DCs to stimulate T cells in the long term. Glucose deprivation or mTOR inhibition can preserve metabolic flexibility and functional OXPHOS in GM-DCs, sustaining their activity at least during 72–96 h and extending their life span. AKT, protein kinase B; CCR7, C-C chemokine receptor type 7; CD, cluster of differentiation; GLUT1, glucose transporter 1; GM-DC, GM-CSF, mouse GM-CSF-induced DCs; HIF1α, hypoxia-inducible factor 1-alpha; HK-II, hexokinase II; IKKε, IkB kinase; IL, interleukin; iNOS, inducible nitric oxide synthase; MHC, major histocompatibility complex; mTOR, mammalian target of rapamycin; NADPH, nicotinamide adenine dinucleotide phosphate; PD-L1, programmed death-ligand 1, Pyr, pyruvate; PPP, pentose phosphate pathway; TBK1, TANK-binding kinase 1; TCA, Tricarboxcylic acid cycle; TNFα, tumor necrosis factor α.

Increasing efforts have been made over the past years to better understand metabolic changes that occur in DCs upon stimulation and how those affect DC functionalities. Resting DCs show a catabolic metabolism and continuously break down nutrients for energy generation and cell maintenance. This metabolic state manifests active OXPHOS, driven by the tricarboxylic acid (TCA) cycle fueled via FAO and glutaminolysis, and is largely regulated by AMPK (13, 41–45), as discussed in the section Metabolic Control of Dendritic Cell Development. Apart from glucose, steady-state DCs use intracellular glycogen to support basal glycolytic demands, which provides metabolic substrates for mitochondrial respiration (46). Upon immunogenic activation, DCs often adopt an anabolic metabolism for the generation of substrates for biosynthesis and cell growth. Activated DCs switch to glycolysis and lactic fermentation that provide energy and additionally reroute glycolytic intermediates into the pentose phosphate pathway (PPP). Moreover, production of nitric oxide (NO), which inhibits the ETC, is induced by some activated DC subsets (Tables 1, 2). The TCA cycle is rewired, leading to accumulation of TCA intermediates that can serve as immunomodulatory signals and support FAS and production of ROS and NO upon DC activation (41–45) (Figure 3). Of note, most of the current knowledge on DC metabolism was obtained using DC-like cells differentiated with GM-CSF from mouse bone marrow *in vitro* (Table 2; GM-DCs), which also contain a significant proportion of macrophage-like cells (12). This DC culture model provides important insights on the basis of metabolic adaptions of DCs after activation but does not allow investigation of different DC subsets, which appear more and more relevant in light of the differential metabolic requirements for their development.

### Increased Glycolytic Activity Determines Inflammatory Dendritic Cell Functions—A Consensus Among Activated DC Subsets?

**Figure 4:**
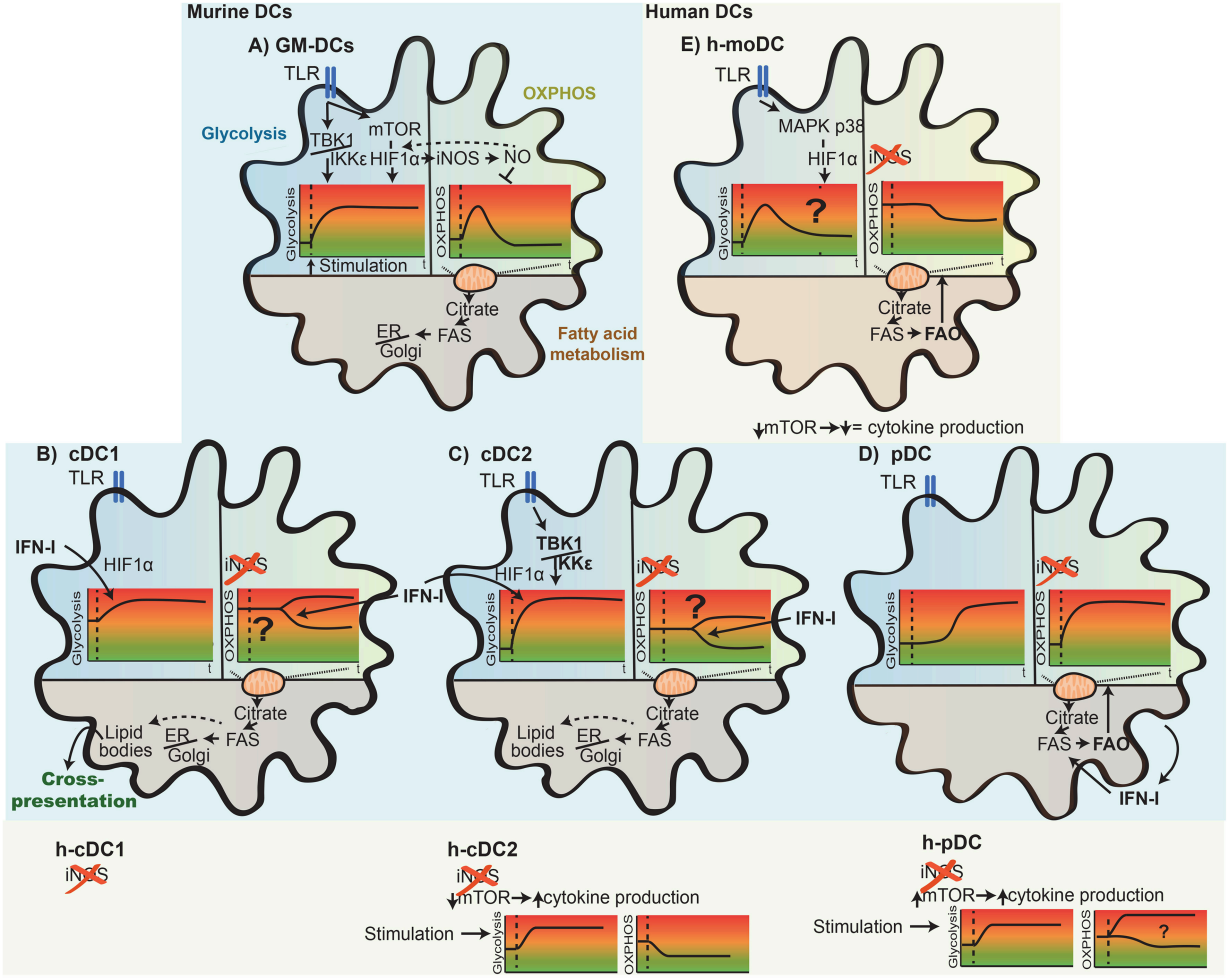
Differential metabolic rearrangement in mouse and human DC subsets upon activation. Depicted here are key adaptions of the main metabolic pathways [glycolysis, OXPHOS (oxidative phosphorylation), and fatty acid metabolism] of DCs upon TLR stimulation. The glycolytic and OXPHOS state of the cells over time (t) is indicated as a schematic representation. In GM-DCs **(A)**, TLR stimulation leads first to induction of glycolysis, and later, mitochondrial OXPHOS is reduced (see also Figure 3). Whereas, this increase in glycolysis is consistently observed after stimulation, differences in the basal glycolytic state, promptness of the glycolytic induction, increased rate, and signaling factors driving these changes in distinct DC subsets are illustrated for naturally occurring mouse and human (h-) cDC1s **(B)**, cDC2s **(C)**, pDCs **(D)**, and human *in vitro*-generated moDCs (h-moDCs; **E**). The impact of TLR stimulation on OXPHOS metabolism among DC subsets likely differs due to the lack of iNOS expression in naturally occurring DCs and h-moDCs. In addition, OXPHOS rearrangements of activated cDCs are context dependent and appear to be down-modulated in splenic cDCs in an IFN-I-dependent manner but remain high in cultured FLT3L-cDCs **(B,C)**. An increase in fatty acid synthesis is generally ascribed to most DC subsets upon stimulation; however, differences in fatty acid use emerge, such as fuel of fatty acid oxidation to drive OXPHOS in h-moDCs and pDCs **(D,E)** or for organelle biosynthesis in GM-DCs, cDC1s, and cDC2s **(A–C)**. In line, fatty acids can accumulate within DCs and form lipid bodies that associate with enhanced cross-presentation potential of cDC1s. Lastly, the thus far reported role of TBK1/IKKε and mTOR/HIF1α regulating cDC, pDC, and h-moDC metabolism and function upon activation is displayed.

An early elevation of glycolysis is a metabolic hallmark of activated DCs and occurs in different mouse DC cultures, human moDCs *in vitro*, and mouse/human DC subsets *in vivo*/*ex vivo* (Figures 3, 4) shortly after pattern recognition receptor (PRR) stimulation with a wide range of pure pathogen-associated molecular patterns (PAMPs) or complex stimuli, such as lipopolysaccharides (LPSs) (13, 47–51), CpG oligodeoxynucleotides (13, 49), poly(I:C) (15, 49), R848/Resiquimod (49, 52), protamine–RNA complexes (pRNA) (53), zymosan (50), Pam_3_CSK_4_/Pam_2_CSK_4_ (49), *Aspergillus fumigatus* (54), *Chlamydia* (55), heat-killed *Propionibacterium acnes* (13), and influenza A virus or rhinovirus infection (52). Interestingly, stimulants such as LPS and zymosan strongly induce upregulation of costimulatory molecules and cytokines, whereas weak activators such as house dust mite (HDM) or zymosan lacking TLR ligands (ZymD) provoke a milder GM-DC maturation profile (56). Importantly, the potency of stimulants inducing GM-DC activation is directly correlated with enhanced degree and maintenance of glycolysis induction (56).

#### Requirement of Glycolysis for Functions of Activated Dendritic Cells

Interrupting the glucose-to-pyruvate pathway significantly impairs DC maturation, upregulation of co-stimulatory molecules, cytokine secretion, and T cell stimulatory capacity in the long term (Figure 3). For example, pharmacological blockade of glycolysis using 2-deoxyglucose (2-DG), genetic deficiency of glycolytic enzymes such as α-enolase (ENO1), or overexpression of lactate dehydrogenase A (LDHA) or pyruvate dehydrogenase kinase 1 (PDK1) (Figure 2) prevents GM-DC maturation and immunogenicity upon stimulation with LPS or *Chlamydia* (13, 47, 49, 57) and can skew GM-DCs toward inducing Th17 and regulatory T cells (Treg) rather than Th1 and Th2 responses (49). In line, natural mouse cDC1s and cDC2s isolated from the spleen decrease expression of co-stimulatory molecules, IL-12 production, and activation of CD4+ and CD8+ T cells when activated by LPS in the presence of 2-DG (49). pRNA-stimulated human blood cDC2s require glycolytic activity for activation, evidenced by TNFα production, CD86, and programmed death ligand 1 (PD-L1) expression (53). Treatment of primary human pDCs with 2-DG upon influenza A virus stimulation also reduces co-stimulatory molecule and type I interferon (IFN-I) expression (52), while another study rather suggests induction of glutamine-fueled OXPHOS upon pRNA stimulation of human blood pDCs (53). However, the effects of inhibition of glycolysis by 2-DG in DCs have to be taken with caution, as 2-DG itself deregulates cytokine expression of human moDCs *in vitro* by activation of the endoplasmic reticulum (ER) stress response via the sensor inositol-requiring protein 1α (IRE1α) (50). In addition, 2-DG can impair the TCA cycle, OXPHOS, and ATP levels, as recently described in macrophages (58).

Other DC functions such as phagocytosis do not seem to be affected by inhibition of glycolysis during stimulation of human moDCs (50). However, reduced endocytic/phagocytic activity in aging mouse spleen cDC1s and DN-DCs [termed merocytic DCs (mcDCs)] and a resulting decline in antigen cross-presentation are linked to mitochondrial dysfunction with decreased basal OCR and ΔΨm as well as enhanced proton leakage and ROS. Importantly, inhibition of ATP synthase by oligomycin or the uncoupling agent carbonyl cyanide 4-(trifluromethoxy)phenyl-hydrazone (FCCP) corroborates the diminished phagocytosis of cDC1s and DN-DCs/mcDCs (22). Moreover, antigen uptake seems to decrease in GM-DCs in hypoxia, when glycolytic activity is increased by HIF1α stabilization, which is also observed in human moDCs after stimulation (47, 50).

In contrast, glucose and enhanced glycolytic activity are required for the ability of DCs to migrate (Figure 3). Independently of stimulation, glucose-deprived GM-DCs show reduced mobility, increased rounded morphology losing dendrites, and impaired oligomerization of CCR7, the chemokine receptor driving DC migration toward LNs. Subsequently, glucose limitation or 2-DG presence prevents migration of GM-DCs as well as splenic CD11c+ cDCs both *in vitro* and *in vivo* (49, 56). In line, HIF1α-deficient GM-DCs, which largely fail to induce glycolysis (see the section Sustained Glycolysis: The Role of HIF1α), display reduced CCR7 levels, and GM-DCs differentiated in hypoxic conditions exhibit elevated migratory potential *in vitro* and *in vivo* that is dependent on HIF1α (14).

Overall, early induction of glycolysis emerges as a general feature of immunogenic activation of most cultured DCs and primary DC subsets and appears necessary for several aspects of their maturation such as upregulation of co-stimulatory surface molecules and cytokine production, despite having no major effects on phagocytosis or antigen uptake. However, DC activation leads to cytoskeletal changes that support increased migratory capacity to migrate toward LNs and T cell zones, which is also affected by early induced glycolysis. Ultimately, in light of those findings, glycolytic increase in DCs upon stimulation is vital for adequate induction of adaptive T cell responses (59) and, hence, regulates immune homeostasis (Figure 3).

### Mechanisms That Control Glycolytic Reprogramming in Activated Dendritic Cells

#### Fuels for Glycolytic Induction Upon Dendritic Cell Stimulation

Extracellular glucose consumption by DCs is required for some aspects of induction of glycolysis, functionality, and survival in activated DCs (13, 56). However, glucose uptake and its effects on DC activation emerge to be time and DC subset dependent. Expression of glycolytic enzymes is not increased in GM-DCs at 4 or 8 h after LPS, HDM, curdlan, or zymosan stimulation (56, 60), when cells already display an enhanced glycolytic activity (56), but is only detectable 18–24 h after stimulation. Moreover, switching GM-DCs from a glucose-containing to a galactose-containing medium, which only supports a low glycolytic rate, 8 h after LPS stimulation actually enhances co-stimulatory molecule expression, IL-12 production, and their potential to activate CD8+ T cells, which is ascribed to deregulation of the mTORC1/HIF1α network (60) (Figure 3). Indeed, increased glycolysis may be preferentially supported by glycogenolysis of intracellular glycogen reserves during the first 6 h post-stimulation of DCs, rather than extracellular glucose (46). GM-DCs activated with LPS or IL-4 during differentiation accumulate intracellular glycogen, which correlates with their enhanced T cell stimulation potential (61). At later stages after GM-DC stimulation, extracellular glucose uptake is enhanced via the upregulation of glucose transporters such as the glucose transporter 1 (GLUT1) (13, 46, 56), and GLUT1 inhibition 24 h after LPS stimulation reduces CD40 and CD86 expression (46). Of note, expression levels of GLUT1 might be a suboptimal readout for its induction or activity. In fact, GLUT1 is translocated from intracellular vesicles to the cell membrane for glucose uptake upon LPS stimulation in macrophages, which does not entirely correlate with mRNA expression levels (62). Moreover, a significant amount of glucose imported from the extracellular environment by activated DCs still appears to be metabolized to glycogen first before entering glycolysis (glycogen shunt; Figure 2) (46). Additionally, upon 6 h pRNA stimulation of primary human blood cDC2s, glycolytic metabolism appears to rely on BCL2 interacting protein 3 (BNIP3)-dependent mitophagy, despite reported 2-DG-sensitive glucose uptake and ENO2 upregulation (53).

#### Early Glycolytic Induction: The TBK1/IKKε/AKT/HK-II Axis

Glycolytic reprogramming upon activation of DCs appears to be largely driven by TANK-binding kinase-1 (TBK1)/IκB kinase-ε (IKKε)/AKT/hexokinase (HK)-II activation in the short term and regulated by AMPK loss and induction of mTOR and/or HIF1α in the long term (Figure 3). TBK1 and IKKε, both non-canonical IκB kinase homologs downstream of TLRs, are activated in GM-DCs within minutes after LPS stimulation, leading to PI3K-independent AKT phosphorylation and association of the rate-limiting glycolytic enzyme HK-II with mitochondria. These events promote glycolytic flux and support early induction of glycolysis in LPS-stimulated GM-DCs as well as in primary mouse spleen cDC2s *ex vivo* (49) (Figure 4). Indeed, early induction of TBK1, AKT, and mTORC1 occurs upon stimulation with potent and weak stimuli, correlating with early increase in glycolytic activity (56). LPS-stimulated human moDCs *in vitro* also enhance HK-II expression and activity in concert with enhanced glycolysis and cytokine production in the long term; however, HK-II induction and glycolysis in this setting appear to rely on HIF1α activity mediated by p38/mitogen-activated protein kinase (MAPK; Figure 4). Nevertheless, this p38/MAPK/HIF1α axis does not seem to be involved in enhanced glycolysis by human moDCs after TLR2/6-mediated activation but relies on TBK1 (51). Notably, HK-II itself can act as a PRR and cause inflammasome activation (63).

#### Glycolytic Reprogramming: AMPK vs. the PI3K/AKT/mTOR Pathway

Loss of AMPK and induction of the PI3K/AKT/mTOR pathway (Figure 1) at longer time points after LPS stimulation of GM-DCs (18–24 h) ultimately lead to upregulation of glycolytic enzymes such as LDHA, pyruvate kinase 2 (PKM2), or phosphofructokinase (PFK), as well as glucose transporters like GLUT1 (13, 56), which depend on glucose availability (60) (Figure 3). Mechanistically, inactivation of AMPK occurs upon LPS stimulation, alleviating mTORC1 inhibition (13, 60). In line, activation of AKT, mTORC1, and mTORC2 declines 18 h after weak stimulation of GM-DCs hand in hand with loss of increased glycolysis activity (56). Enforced AMPK activation or inhibition/loss can prevent or foster GM-DC maturation, respectively (13, 33), associating active AMPK with diminishing proinflammatory DC functions (59). Human pRNA-activated cDC2s downregulate AMPKα1 levels, which appears to be dependent on mitophagy in this system (53). A reduction in glycolysis and activation of GM-DCs upon early inhibition of glycogenolysis also associate with a rapid drop in intracellular ATP and AMPK activation (46). Inhibition of mTOR/mTORC1 blunts glucose consumption, lactate production, upregulation of glycolytic enzymes/glucose transporters, and increased extracellular acidification rate (ECAR) in GM-DCs 20 h or longer after LPS stimulation (60, 64). Hence, mTOR activation appears to control DC activation, especially maintaining it for prolonged periods of time (43) (Figure 3). Indeed, ectopic AKT/PKB activation, which sustains mTOR activation, enhances co-stimulatory molecule expression and cytokine secretion in human pDCs (32). Also, mTOR signaling is essential for induction of IFN-I responses of (primary) mouse and human pDCs (65). In concert, rapamycin treatment of anti-CD40-stimulated GM-DCs *in vitro* or IL-4-treated spleen CD11c+ DCs *in vivo* downregulates co-stimulatory molecules/cytokines and promotes activation of Tregs, but not allogeneic CD4+ T cells (30, 66).

Nevertheless, sustained mTOR signaling may also be detrimental for proinflammatory DC functions (23, 41, 59, 67). For example, knockdown or pharmacological inhibition of mTOR enhances life span, prolongs the expression of co-stimulatory molecules, cytokine production, and promotes T cell stimulatory activity of LPS-stimulated GM-DCs (64, 68, 69) (Figure 3). Indeed, mTOR promotes NO production by activated GM-DCs, which limits their mitochondrial energy metabolism, while mTOR inhibition restores the metabolic flexibility of those cells in the long term (68) (Figures 3, 4). However, loss of the negative mTORC1 regulator TSC1 in mouse DCs causes impaired cytokine production and antigen presentation upon TLR4 stimulation (36). mTORC1 inhibition in human CD1c+ cDC2s enhances proinflammatory cytokine production upon stimulation with various agents but has the opposite effect on LPS-stimulated human moDCs. Those contrasting effects are ascribed to differential activation of NFκB upon mTORC1 blockade, which increases in LPS-stimulated CD1c+ cDC2s but remains unchanged in moDCs (2). A spatiotemporal model to integrate the ambiguous roles of mTOR regulating DC functions has been proposed (23).

#### Sustained Glycolysis: The Role of HIF1α

HIF1α stabilization is also involved in enhanced glycolytic activity of GM-DCs and human moDCs upon stimulation *in vitro* and of natural mouse cDCs *in vivo* (15, 47, 51) (Figures 3, 4). Many glycolytic genes are HIF1α targets and are downregulated in DCs upon HIF1α loss, such as GLUT1 and LDHA (47, 50, 54, 60). Moreover, only potent GM-DC stimulation that leads to long-term induction of glycolysis causes HIF1α stabilization and induction of its target genes, while weak activation fails to do so (56). In line, GM-DCs in the steady state express higher MHCII and co-stimulatory molecule levels in hypoxic conditions (14). LPS stimulation of GM-DCs in hypoxia compared with normoxia further elevates HIF1α activation, glucose consumption, glycolytic enzyme expression, and lactate and ATP production, enhancing GM-DC activation (47). Similar effects are also observed upon *in vitro Aspergillus fumigatus* stimulation of human moDCs in hypoxia *in vitro* (54). Inhibition or loss of HIF1α in GM-DCs or human moDCs prevents the increase in glycolytic rate and upregulation of glycolytic genes upon LPS or *Aspergillus fumigatus* stimulation and reduces co-stimulatory molecule expression, proinflammatory cytokine production (including IL-12), and CD4+ T cell stimulatory capacity in the long term (47, 51, 54). However, LPS-stimulated HIF1α-deficient GM-DCs show enhanced IL-12 expression and CD8+ T cell activation (60). Hence, further efforts will be necessary to clarify the exact role of HIF1α on DC functions. Nevertheless, spleen CD11c+ MHCII+ cDCs of mice lacking HIF1α in CD11c-expressing cells also fail to induce higher glycolysis and display reduced immunogenicity 14–18 h after poly(I:C) stimulation. However, some of those effects might be ascribed to elevated death of HIF1α-deficient spleen cDCs (15). Importantly, HIF1α can be induced or stabilized by many other mechanisms apart from mTORC1 or hypoxia, such as glucose withdrawal (60), which might differentially influence the effects on immunogenic DC activation. Moreover, HIF1α can be activated by intracellular pyruvate or lactate produced by glycolysis (70, 71). Indeed, the timing of HIF1α stabilization occurring in human moDCs 4 h after LPS or zymosan stimulation trails the immediate increase in glycolysis (50). Notably, weakly stimulated GM-DCs do not accumulate HIF1α while still inducing early glycolysis, in contrast to strongly activated GM-DCs that stabilize HIF1α and maintain high glycolytic activity at later stages (56). Taken together, HIF1α is implicated in the maintenance rather than in the early induction of glycolysis after DC stimulation (50) (Figures 3, **4**) and appears to partially depend on glucose availability (60).

#### Extracellular Cues Influencing Glycolytic Metabolism of Activated Dendritic Cells

Signals in the microenvironment can strongly influence DC function via modulating their glucose metabolism. For example, the anti-inflammatory cytokine IL-10 inhibits the LPS-mediated increase in glycolysis and GM-DC maturation likely via maintaining active AMPK (13), and IL-10-deficient GM-DCs display higher levels of the glycolytic enzyme ENO1 (57). Similarly, IL-10 loss in macrophages causes enhanced glycolytic reprogramming upon LPS stimulation, which is ascribed to mTORC1 inhibition by autocrine IL-10 via signal transducer and activator of transcription 3 (STAT3) and DNA damage inducible transcript 4 (DDIT4). Notably, they also accumulate dysfunctional mitochondria due to reduced autophagy independent of NO (62).

Metabolic reprogramming of mouse spleen cDCs may rely on type I IFNs in concert with PRR signaling, as IFNα/β receptor (IFNAR)-deficient cDCs fail to elevate glycolytic activity after poly(I:C) stimulation *in vivo* while maintaining active OXPHOS (15), and mouse pDCs from FLT3L-DC cultures increase their OCR and ECAR upon 24 h exposure to IFNα (72) (Figure 4). However, IFNα treatment or IFNAR inhibition in primary human blood pDCs *ex vivo* does not affect induction of glycolysis after stimulation with influenza A virus (52).

Last, exogenous metabolites such as fatty acids or lactate are sensed by DCs, leading to an adaption of their metabolism and functions [reviewed in Pearce and Everts (41)], such as lactate-mediated effects on HIF1α (70, 71, 73). For example, the short-chain fatty acid butyrate can prevent maturation and glycolytic reprogramming of human moDCs upon LPS stimulation, driving them to induce Tregs (74).

### Fatty Acid Synthesis and ER Stress During Dendritic Cell Activation

Generation of TCA cycle intermediates regulates function and *de novo* FAS upon DC stimulation. Indeed, while glycolysis-derived ATP appears to be dispensable for early GM-DC activation, incorporation of pyruvate into the mitochondrial TCA cycle is essential, as knockdown of the mitochondrial pyruvate carrier MPC-1 (Figure 2) limits GM-DC maturation and cytokine production (49). Accumulation of TCA intermediates such as citrate, succinate, and fumarate in stimulated DCs contributes to the regulation of inflammatory responses as well as cytokine production (45, 75). Additionally, citrate escaping the mitochondria serves as an important substrate for protein acetylation, nicotinamide adenine dinucleotide phosphate (NADPH) production, and, importantly, cytosolic FAS in activated DCs (49, 75) (Figures 2, 3). In addition, knockdown of the PPP enzyme glucose-6-phosphate dehydrogenase (G6PDH) reduces LPS-induced maturation of GM-DCs (49). The PPP produces ribose 5-phosphate (R5P), a precursor for biosynthesis of nucleotides, and NADPH, which is needed for production of ROS and NO as well as for cytosolic FAS (Figure 2).

*De novo* FAS and accumulation of phospholipids increase upon GM-DC stimulation with LPS (49) and after activation of *in vitro* bone marrow-derived cDC1-like cells (iCD103; Table 2) with LPS, CpG, and *Mycobacterium bovis* Bacille Calmette Guerin (BCG) (76) (Figure 4). Indeed, accumulation of intracellular fat in LPS- or IL-4-stimulated GM-DCs correlates with enhanced T cell activation capacity (61). FAS also leads to increased lipid storage in lipid bodies (LBs) in GM-DCs (49), organelles composed of a core of neutral lipids such as cholesteryl esters or triglycerides (TAG) surrounded by a single layer of phospholipids (77). Notably, intracellular LB formation associates with induction of cross-presentation potential in GM-DCs, FLT3L-DCs, and mouse spleen cDCs that is at least partially dependent on inflammasome activation or IFNγ-induced protein immunity-related GTPase family member m3 (Irgm3) (78, 79). Accordingly, the specialized cross-presenting CD8+ cDC1 subset (Table 1) in the spleen harbors more LBs than CD8– cDCs (79). Human and mouse liver DCs with high lipid content are more potent activators of NK, CD4+, and CD8+ T cells, which is reduced by inhibition of FAS (80). In line, FAS blockade in GM-DCs by knockdown of the mitochondria–cytosol citrate shuttle citrate transport protein (CTP) or by the FASN or ACC inhibitors C75 and TOFA (Figure 2) prevents LPS-induced activation and proinflammatory functions of GM-DCs (49). However, non-activated GM-DCs or human moDCs differentiated in the presence of TOFA show high levels of ER stress, ERK and AKT signaling, and PPARγ expression, linked to enhanced DC immunogenicity and T cell priming (7). In the iCD103 culture system that rather represents cDC1-like DCs (Table 2), deficiency in ACC1 or 2 or their inhibition by TOFA does not affect co-stimulatory surface marker expression and their inflammatory cytokine profile upon CpG or *Mycobacterium bovis* BCG stimulation. T cell priming capacity or *in vivo* mycobacterial control of iCD103 DCs also remains unaffected by interference with FAS (76). Of note, FAS impairment in iCD103s also results in enhanced uptake of extracellular fatty acids, which might represent a compensatory mechanism for fatty acid generation. Nevertheless, the actual role and subsequent usage of fatty acids produced by DCs appear to be dependent on the context and DC subsets (Figure 4). For example, *de novo* synthesized fatty acids provide building blocks for expansion of the Golgi apparatus and the ER in LPS-stimulated GM-DCs and are ultimately required for activated DCs to produce and secrete large amounts of cytokines, which can lead to ER stress and the unfolded protein response (41, 49). Liver DCs containing high amounts of lipids have an increased ER stress, and its blockade reduces their ability to induce immune responses (80). Indeed, ER stress can enhance IL-23 production in zymosan-stimulated human moDCs via IRE1α and X-box binding protein 1 (XBP1) (50). In contrast, in mouse pDCs sorted from FLT3L-DC cultures, an increase in ECAR late after CpG or IFNα stimulation associates with enhanced FAS, which, in this setting, serves as a source of fatty acids for FAO to maintain high OXPHOS levels (72) (Figure 4).

Overall, regulation of ER stress and lipid metabolism in activated DCs can notably influence their function to release cytokines and to present antigen (41, 81), and further efforts will be needed to understand the precise functions in different settings. In that regard, the importance of *de novo* FAS and lipid accumulation in tolerogenic or dysfunctional DCs in cancer is discussed in the section Lipid Accumulation and Dendritic Cell Dysfunction in Cancer.

### Mitochondrial Energy Generation Regulating Dendritic Cell Activation—Specific to Dendritic Cell Subsets and the Context

#### Mouse GM-CSF Dendritic Cell Cultures

Development of natural DCs largely relies on FAO to fuel OXPHOS (see the section Metabolic Control of Dendritic Cell Development). However, in cultured GM-DCs, mitochondrial energy metabolism is dramatically reduced upon immunogenic stimulation in the long term (13) (Figures 3, 4). Indeed, the FAO inhibitor etomoxir, the glutaminolysis inhibitor 6-diazo-5-oxo-L-norleucine (DON), or glutamine deprivation has no effect on GM-DC maturation upon LPS stimulation (46, 49). Furthermore, GM-DCs display irresponsiveness to ETC inhibitors and exhibit decreased OCR and ΔΨm 18 or 24 h post-LPS stimulation, which is independent of PI3K/AKT signaling (13, 48). The production of NO via the enzyme inducible NO synthase (iNOS) is central to the collapse of OXPHOS of activated GM-DCs in the long term and their functions (48) and was recently reviewed (82). In brief, NO is induced in GM-DCs within 6 h after LPS stimulation, and their enhanced glycolytic rate becomes NO dependent about 9 h after stimulation, when OXPHOS declines (49). Stabilized HIF1α enhances NO generation by increasing the expression of iNOS, which, in turn, leads to the inhibition of prolyl hydroxilases (PHDs) that label HIF1α for degradation. This positive loop causes NO accumulation, which leads to nitrosilation of some ETC complexes and inhibits their functionality (48, 60, 82) (Figures 3, 4). A small proportion of mouse moDCs induced by *Listeria monocytogenes* infection also display a comparable NO-mediated inhibition of OCR late after stimulation that is compensated by enhanced glycolysis (48). Based on those and other studies in tolerogenic DCs (see the section DC Metabolism in Tolerance), anabolic metabolism, and glycolysis are generally associated with immunogenicity of DCs, while catabolic metabolism and active mitochondrial respiration, regulated via AMPK/PGC1α, are related to tolerogenicity of DCs (41, 43, 83).

However, several pieces of evidence point toward a potential role of mitochondrial energy metabolism and functional OXPHOS in immunogenic, activated DCs. Indeed, ΔΨm and OCR are actually increased in GM-DCs in the short term up to 6 h after LPS stimulation before iNOS becomes expressed (Figure 4), which is prevented by 2-DG (48, 49), and weak stimuli like HDM or ZymD do not reduce mitochondrial respiration 18 h post-activation (56). Moreover, decreased mitochondrial abundance is usually not associated with NO-mediated OXPHOS inhibition upon GM-DC activation (13), and 24 h LPS-activated GM-DCs or mouse moDCs fully restore their mitochondrial respiratory profile when NO production is diminished (48). Also, ENO1 loss causes a profound dysregulation of mitochondrial morphology in short-term (2 h) *Chlamydia*-stimulated GM-DCs associated with a drop of intracellular pyruvate levels and enhanced cell death (57). Additionally, antiviral responses of DCs promoted by cytoplasmic RNA sensor RIG-I-like receptor (RLR) signaling depend on the mitochondrial localization of the antiviral signaling protein (MAVS), which requires active ΔΨm (41).

These observations suggest that mitochondrial energy generation contributes to DC activation in certain settings. Indeed, deficiency or inhibition of iNOS in LPS-activated GM-DCs maintains active OXPHOS and even enhances aspects of DC activation, such as CD8+ T cell stimulation and CD86 and MHC molecule expression in the long term (48) (Figure 3). The presence of the mTORC1 inhibitor rapamycin attenuates NO production and ameliorates the decrease in mitochondrial-dependent OCR in activated GM-DCs (60, 68). The maintenance of functional OXPHOS permits the cells to use FAO and glutaminolysis for energy generation (68). Also, the culture of LPS-activated GM-DCs in galactose enhances OCR, while ECAR levels plummet (60). Indeed, in the long term, rapamycin-treated or galactose-cultured activated GM-DCs display a prolonged life span together with extended co-stimulatory molecule and IL-12 expression that leads to more potent activation of CD8+ T cells, which is at least partially dependent on suppression of HIF1α/iNOS signaling (60, 64, 68) (Figure 3).

#### Natural Mouse and Human Dendritic Cell Subsets

Crucially, contrary to cultured GM-DCs, most DC subsets present in lymphoid organs do not express detectable levels of iNOS, foremost naturally occurring cDC1s and cDC2s, as well as cultured human moDCs (82) (Tables 1, 2 and Figure 4). In line, mitochondrial energy metabolism and OXPHOS remain intact in *in vitro* LPS- or zymosan-stimulated human moDCs (50). Also, splenic mouse cDC1 and cDC2 increase their ECAR shortly after *in vivo* LPS stimulation (49); however, notably, they do not display any differences in the ECAR/OCR ratio 24 h after *ex vivo* LPS stimulation (48) (Figure 4). Uptake of dead cell material and cross-presentation potential of unstimulated natural mouse spleen cDC1s and DN-DCs/mcDCs (Table 1) are diminished upon abrogated mitochondrial function caused by aging or ETC inhibition (22). Conversely, 14 h *in vivo* poly(I:C) stimulation reduces ΔΨm and OCR of total spleen cDCs, which is prevented by IFNAR deletion (15) (Figure 4), suggesting an additional context-mediated mechanism. In the same study, maintenance of mitochondrial energy metabolism and reduction in ECAR upon poly(I:C) stimulation by HIF1α loss in spleen cDCs reduce their T cell activation potential. However, this effect is ascribed to unbalancing cellular metabolism leading to enhanced ROS production, lower ATP levels, and increased cell death (15). Nevertheless, in 6 h-stimulated human blood cDC2s, mitochondrial morphology and dynamics are altered, the OCR is strongly reduced, and BNIP3-dependent mitophagy is triggered, which appears necessary for glycolytic activity and activation (53).

Notably, pDCs appear to show distinctive rewiring of their mitochondrial energy metabolism in different settings (Figure 4). While human pDCs mildly decrease their OCR after 24 h *ex vivo* influenza or rhinovirus infection (52), they elevate OXPHOS 6 h post-pRNA stimulation, which appears to be mediated by autophagy-induced glutaminolysis (53). Importantly, the induction of mitochondrial energy metabolism in human pDCs is required for the production of IFNα, CD80, and PD-L1 expression (53). Mouse pDCs sorted from FLT3L-DC cultures enhance mitochondrial pyruvate import and FAO that fuel elevated OXPHOS 24 h post-CpC stimulation. This effect is due to IFN-I induction, with IFNα itself promoting FAO via PPARα (72), in contrast to mouse spleen cDCs where IFNAR deficiency maintains high OCR (15) (Figure 4).

Hence, no general conclusion can be reached as to the importance and function of mitochondrial energy metabolism, OCR, and OXPHOS in activated DCs, and it appears context and DC subset dependent (Figure 4). Metabolic flexibility of activated DCs to switch their carbon source for ATP generation from glucose to galactose, glutamine or fatty acids would be of benefit in DC function and indeed, prevention of OXPHOS collapse and metabolic plasticity enhance DC survival and activation upon glucose deprivation and mTOR or iNOS inhibition (48, 60, 68). In the future, it will be interesting to determine the influence of the microenvironment in which DCs are activated. Not only nutrient or oxygen availability but also other environmental factors can strongly influence DC metabolism, such as extracellular lactate, fatty acids (41), the TCA intermediates citrate, succinate, and fumarate (45, 75), as well as IL-10 (13, 57) or IFN-I (15, 52, 72), as discussed in the section Extracellular Cues Influencing Glycolytic Metabolism of Activated Dendritic Cells. Moreover, NO produced by neighboring cells can cause HIF1α stabilization and trigger a cellular loop in DCs, leading to a glycolytic switch (60, 68).

Development and maintenance of different DC subsets display differential metabolic requirements (discussed in the section Metabolic Control of Dendritic Cell Development), which will likely reflect on their metabolic reprogramming upon activation. Considering that different DC subsets specialize on distinct functions (Table 1), their metabolic requirements to exert those tasks might differ, as suggested in a recent study (53). Moreover, a fine regulation of OXPHOS activity, such as reported in the case of supercomplex assembly in macrophages (84, 85), may also have a functional effect on DCs.

## Dendritic Cell Metabolism in Tolerance

DCs contribute to the maintenance of immunological tolerance in order to prevent hyperactivation of the immune system and subsequent autoimmune diseases. Generally, such tolerogenic DCs arise in the steady state during uptake of (self-) antigen in the absence of danger signals, upon sensing of anti-inflammatory cytokines/factors, and during various pathological states, including cancer, due to tolerizing signals (86, 87). Tolerogenic or semimature DCs can be identified by upregulation of regulatory surface molecules or receptors such as PD-L1 and tolerogenic cytokines IL-10, IL-27, and TGFβ, leading to induction of Treg activation at the expense of effector T cells (83, 86). Much of the functionality of tolerogenic DCs is intertwined with metabolic activity, such as lipid accumulation or catabolism of amino acids [tryptophan (Trp) and arginine (Arg)].

### Metabolic State(s) and Their Regulatory Cellular Pathways in Tolerogenic Dendritic Cells

#### Metabolic Adaptions of Tolerized Dendritic Cells

Our understanding of energy metabolism of tolerogenic DCs is largely based on observations in human moDC cultures treated with vitamin D3 or D2 (VitD3 or VitD2), dexamethasone (DEX), and/or resveratrol (83, 88–91). Resveratrol is a plant-derived polyphenol that induces regulatory properties in mouse and human DCs, preventing their maturation and immunogenic activation (92, 93). Glucocorticoid receptor engagement by DEX modulates many aspects of DC maturation, including antigen presentation and cytokine production, leading to a tolerant phenotype (83, 94, 95). VitD3 skews DC functionality toward an inhibitory phenotype inducing Tregs and enhancing expression of inhibitory receptors (96, 97).

Tolerogenic human moDCs, generated either by treatment with DEX+VitD3 for 48 h or 1,25-dihydroxyvitamin D3 [1,25(OH)2-VitD3, the active form of VitD3] for 24 h, exhibit enhanced catabolism and metabolic plasticity, increased expression of genes involved in OXPHOS, glycolysis/glucose metabolism, and FAO in concert with higher mitochondrial respiration (OCR) and glycolytic activity (ECAR) than untreated moDCs (88, 89). Intriguingly, LPS stimulation of DEX+VitD3-tolerized moDCs slightly decreases their OXPHOS capacity; however, their glycolytic capacity drops to levels of immunogenic LPS-stimulated DCs, which are, in this study, lower than those of untreated moDCs (88). Functionally, while MHCII expression of LPS-stimulated immunogenic moDCs is sensitive to glycolysis inhibition, LPS-stimulated DEX+VitD3-tolerogenic moDCs remain unaffected. DEX+VitD3-tolerogenic moDCs increase their MHCII levels upon inhibition of FAO instead. In line, FAO inhibition rescues the ability of DEX+VitD3-tolerogenic moDCs to induce expression of activation markers on CD4+ T cells upon LPS stimulation (88). Moreover, in the context of melanoma, a Wnt5a/β-catenin and PPARγ pathway induces FAO and a tolerogenic indoleamine 2,3-dioxygenase (IDO)-producing and Treg-activating phenotype in DCs (98). In contrast, in moDCs tolerized by DEX+VitD2, immunogenic stimulation induces even higher glycolysis/cellular LDH activity than in activated moDCs (91). Nevertheless, the maintenance of tolerogenic features of both 1,25(OH)2-VitD3-treated and (re-stimulated) DEX+VitD2-treated moDCs relies on glycolysis, and their tolerogenic phenotype is abrogated by 2-DG treatment (89, 91). Notably, levels of FAO are unaltered in 1,25(OH)2-VitD3-treated vs. control moDCs, and FAO inhibition by etomoxir does not affect their tolerogenic hallmarks (89). Accumulation of pyruvate during glycolysis may partially cause the concomitant increase in OXPHOS in those tolerogenic moDCs in concert with elevated OXPHOS-related gene expression (89). Those results indicate a metabolic plasticity and responsiveness of tolerogenic moDCs, which display a very active metabolism, despite showing differential dependencies on glycolysis vs. FAO/OXPHOS. Those controversies may be due to the different experimental settings, presence or absence of immunogenic stimulation, and the fact that 1,25(OH)2-VitD3 has stronger effects on OXPHOS, lipid, and glucose metabolism of tolerogenic moDCs than DEX (99). Nevertheless, tolerogenic DCs appear to rely less on glycolysis than LPS-activated immunogenic DCs for their functionality and, as they largely upregulate functional OXPHOS, might be able to adapt their metabolism depending on the context. However, those conclusions are solely based on cultured human moDCs, and the metabolism of other tolerized DC subsets in complex *in vivo* settings largely remains to be investigated.

#### AMPK and mTOR Pathways Influence Tolerogenicity of Dendritic Cells

The tolerogenic status of DCs is also influenced by a balance of the nutrient-sensing pathways AMPK and mTOR, which appear to be equally context dependent as for immunogenic stimulation of DCs. Inflammatory activation of DCs involves enhanced glycolytic activity and anabolic metabolism compared to immature DCs that largely appear to be controlled by mTOR signaling (see the section Mechanisms That Control Glycolytic Reprogramming in Activated Dendritic Cells), and, despite controversial findings (64, 68), mTOR inhibition by rapamycin can cause DC tolerization (30, 66). Accordingly, DEX or resveratrol treatment of macrophages can block iNOS expression and NO generation (100, 101), whose upregulation associates with LPS-activated GM-DCs (82), while VitD3 had varying effects (102). Indeed, an axis involving AMPK, PGC1α, and PPARγ is suggested to control tolerogenicity of DCs, largely by preventing biosynthetic metabolic adaptions or pathways driving immunogenic DC activation such as mTOR (41, 43, 59, 83). This concept is founded on the observations that tolerogenic DCs show enhanced mitochondrial respiration and that AMPK activation favors catabolic metabolism, FAO, and OXPHOS, largely via PPARγ and the mitochondrial biogenesis inducer PGC1α (41, 43, 59, 83). Indeed, DEX+VitD3- and 1,25(OH)2-VitD3-treated human moDCs upregulate AMPK activity and signaling (88, 89), human cDC2s (53) and GM-DCs reduce AMPK activation upon pRNA or LPS exposure, and the inhibitory effect of IL-10 on LPS-mediated maturation of mouse GM-DCs appears to be AMPK dependent (13). Further, AMPKα1-deficient LPS-stimulated GM-DCs show augmented proinflammatory features such as enhanced co-stimulatory molecule expression and CD40 signaling, increased IL-6 and TNFα, but decreased IL-10 production and skewing of CD4+ T cell activation toward a Th1 and Th17 phenotype (33). The AMPK inducer 5-aminoimidazole-4-carboxamide ribonucleotide (AICAR) is equally potent in blocking glucose consumption by LPS-stimulated GM-DCs as 2-DG (13) and AMPK activation after uptake of dead cells induces autophagy, tolerogenic properties, and reduced anti-tumor immune responses (103). Intriguingly, several studies also implicate VitD3, resveratrol, and DEX in enhancing AMPK activation in various other settings and cell types (104–110). Moreover, resveratrol treatment promotes OXPHOS and mitochondrial biosynthesis in mice and humans via mechanisms similar to AMPK, such as activating the histone deacetylase Sirtuin 1 and augmenting PGC1α expression (90, 111). Loss of the PGC1α targets PPARγ or nuclear factor erythroid 2-related factor 2 (NRF2) enhances DC maturity and proimmunogenic functionality (41).

However, the precise role of balanced mTOR/AMPK signaling in tolerogenic DCs remains controversial. Indeed, the PI3K/AKT/mTOR axis is reported to be vital for tolerogenic features of moDCs, independent from AMPK (89, 91). Human restimulated DEX+VitD2-tolerized moDCs strongly upregulate mTOR phosphorylation and signaling compared to non-tolerized controls (91). PI3K or mTOR inhibition (by LY294002 or rapamycin, respectively) enhances MHC and co-stimulatory molecule expression and reduces co-inhibitory molecules as well as the IL-10/IL-12p70 expression ratio by 1,25(OH)2-VitD3-treated and DEX+VitD2-treated moDCs without or after immunogenic activation. Induction of CD4+ and CD8+ T cell proliferation and IFNγ production is also enhanced by mTOR inhibition in both tolerogenic human moDC cultures (89, 91). Importantly, in this setting, AMPK activation by AICAR is ineffective in altering the tolerogenic phenotype of 1,25(OH)2-VitD3-treated moDCs (89). Moreover, the context dependence of cellular metabolism associated with active mTOR signaling is highlighted by a recent study of allergic airway inflammation in mice harboring mTOR-deficient CD11c-expressing cells (31). There, HDM exposure induces the generation of lung CD11c+ MHCII+ CD11b+ DCs that depend on macrophage CSF (M-CSF) and, hence, likely represent moDCs (Table 1). Upon loss of mTOR, those induced CD11b+ DCs show enhanced expression of CD80 and CD86 co-stimulatory molecules and skew the HDM-mediated Th2-polarized allergy toward a neutrophilic Th17-mediated lung inflammation. Moreover, mTOR-deficient CD11b+ DCs accumulate fatty acid metabolites, and FAO inhibition by etomoxir diminishes their activated phenotype (31). Those observations suggest anti-inflammatory/tolerizing effects of mTOR associated with inhibition of FAO that, in turn, appears functionally important for an activated state and Th17 polarization capacity of lung CD11b+ inflammatory DCs in allergic airway inflammation.

In summary, research on primary DC subsets in settings of immune tolerance, additional to tolerized DC cultures, will be needed to advance our knowledge on tolerogenic DC metabolism.

### Lipid Accumulation and Dendritic Cell Dysfunction in Cancer

The role of lipid metabolism for immunogenic and tolerogenic DC function is ambiguous. Although lipid accumulation in DCs seems to support immunogenic immune responses and cross-presentation (78, 79) (see the section Fatty Acid Synthesis and ER Stress During Dendritic Cell Activation), it also associates with DC dysfunction in tumor settings. Tumor-associated DCs accumulate high amounts of cytosolic lipids in both mice and humans. Lipid-laden DCs isolated from tumor-bearing mice exhibit defective T cell stimulation ability due to altered antigen processing and presentation (112). The aberrant lipid accumulation in DCs is fostered by yet-unknown factors secreted by tumor cells and mediated by macrophage scavenger receptor 1 (Msr1) on DCs (112), a receptor that binds primarily modified lipoproteins (113). Inhibition of Msr1 or blockade of FAS with TOFA restores lipid content and DC immunogenicity, indicating that enhanced lipid uptake, FAS, or a combination impairs DC-mediated antitumor immunity. Interestingly, this effect is observed in cDC1s and cDC2s but not in pDCs (112), which might be a reflection of the different functions and/or metabolic pathway usage among DC subsets *in vivo* (Table 1 and Figure 4). Indeed, CD103+ cDC1s from draining LNs (dLNs) of tumor-bearing mice accumulate more LBs compared to the CD103– DC counterparts, which substantially reduces their ability to cross-present antigens (114). Cross-presentation plays a central role in the generation of efficacious anticancer CD8+ cytotoxic T cell responses (115), and these data provide a metabolic explanation for the impaired ability of tumor-infiltrating DCs to induce potent antitumor adaptive responses.

The differential effect of lipid accumulation in DCs seen in tumor settings may be due to accumulation and/or signaling by modified lipid species. For instance, tumor-derived factors act on DCs activating liver X receptor (LXR)-α signaling, whose natural ligands are oxidized cholesterol (oxysterols), and reduce the expression of CCR7, inhibiting their migration to the dLNs (116). Consistently, LXR-α/LXR-β-deficient GM-DCs show impaired migration in response to the CCR7 ligands CCL19 and CCL21, and this response is partially dependent on the LXR target CD38, a molecule that is linked to leukocyte trafficking (117). Oxidized lipids contained in tumor-associated DCs also affect cross-presentation (118). Accumulation of oxidized polyunsaturated fatty acids, cholesterol esters, and TAG impairs cross-presentation without affecting the presentation of endogenous antigens. Notably, the accumulation of non-oxidized lipids does not alter cross-presentation, supporting the idea that it is not the mere storage of lipids but the accumulation of modified lipids that alters DC function (114, 118). Consistent with these observations, tumor-derived factors trigger lipid peroxidation in tumor-associated DCs, which activates the ER stress response mediated by IRE-1α and its target XBP1. XBP1 activation, in turn, induces a lipid biosynthetic program that results in the accumulation of LBs and blunted antigen presentation, leading to a reduced ability to control tumor growth (119). Regarding the mechanisms by which LBs and modified lipids could impair cross-presentation, oxidatively truncated TAG accumulate on the surface of LBs and bind the heat shock-induced chaperone heat shock protein 70 (HSP70). As a result of this interaction, peptide–MHCI complexes do not traffic to the cell surface and rather accumulate in lysosomal/late endosomal compartments (114), although the mechanism by which HSP70 controls antigen cross-presentation remains to be elucidated.

Taken together, these data illustrate mechanisms by which capabilities of DCs are suppressed in tumors through modification of their lipid metabolism, either by secreted factors or indirectly by an altered tumor microenvironment. Tumor-associated DCs exert their functions in a tissue where glucose is scarce due to the high glycolytic rates of tumor cells (120), and the inability to adopt a glycolytic metabolism can impair DC effector functions (see the section Metabolic Rearrangements Upon Immunogenic Dendritic Cell Stimulation). Alternatively, tumor-derived factors can enforce FAO and OXPHOS in DCs and promote accumulation of lipids, which can, in turn, inhibit secretion of proinflammatory cytokines and antigen cross-presentation, respectively (98, 112, 119). Nonetheless, it remains unanswered why and how tumor-associated DCs accumulate high amounts of lipids. Some reports indicate that lipid accumulation is due to activation of a lipogenic program (119), while others suggest increased lipid uptake (112). Moreover, tumor cells also secrete other factors to the local milieu that act on tumor-infiltrating DCs and support the acquisition of a tolerogenic phenotype such as adenosine (121) or lactate (70, 71, 73). Thus, the metabolic reprogramming of tumor-associated DCs can contribute to tumor progression.

### Amino Acid Metabolism and Tolerizing Dendritic Cell Functions

Catabolism of the essential amino acid Trp is critical in balancing inflammation and tolerance. Trp is metabolized by the enzyme IDO1, generating kynurenine (Kyn) in a process that consumes oxygen (122). This enzyme is highly expressed by tumor cells and exploited as a mechanism for immune evasion (123). IDO1-mediated Trp catabolism promotes local immunosuppression by two means: (1) Trp starvation limits T cell proliferation by impairing the T cell cycle machinery (124, 125), and (2) Kyn products induce T cell apoptosis (126), inhibit T cell cytotoxicity via downregulation of T cell receptor (TCR) CD3 ζ-chain (127), and induce differentiation of Tregs (127, 128). Notably, a subset of tumor-associated pDCs that accumulate in tumor-draining LNs (tdLNs) express IDO and mediate antigen-specific T cell anergy, contributing to tumor progression (129, 130). Cytokines such as IFNγ and TGFβ (131–134) and immunosuppressive drugs such as DEX (131) induce IDO in pDCs. Remarkably, cytotoxic T-lymphocyte-associated protein (CTLA)-4-expressing Tregs bind B7 family receptors on pDCs also triggering IDO1 expression (132, 133). This bidirectional conditioning also happens upon glucocorticoid-inducible TNF receptor-related protein (GITR) ligand (GITRL) engagement by GITR, expressed by Tregs and pDCs, respectively, inducing IDO1 expression via activation of the IKB–IKKα non-canonical NFkB pathway in pDCs in an IFNα-dependent manner (131). This crosstalk would establish a positive feedback loop to favor long-term immunosuppression. DEX induces this tolerogenic pathway by concomitant upregulation of GITR on CD4+ T cells and GITRL on pDCs (131). DEX treatment is a frequently used treatment to tolerize human moDCs *in vitro* (see the section Metabolic Adaptions of Tolerized Dendritic Cells), which often display high FAO and OXPHOS rates (88, 89). Therefore, one could hypothesize that FAO and IDO1 activities collaborate in establishing a tolerogenic program in DCs. Indeed, an oxidative metabolic profile adopted by tolerogenic DCs supports IDO1 function, providing a direct link between FAO and tolerogenic DC responses *in vivo* (98).

Arg is another amino acid that has a central immunomodulatory role. In immune cells, Arg is metabolized by iNOS under inflammatory conditions to generate L-citruline and NO (135), the latter being associated with activated GM-DCs (82). Alternatively, Arg can be metabolized by arginases 1 and 2 (Arg1 and 2) to produce ornithine, a precursor for polyamines that can support tumor cell proliferation (135, 136). Notably, tumor-infiltrating DCs act as Arg sinks, contributing to local Arg depletion and indirectly inhibiting T cell antitumor responses (137). Additionally, Arg1-dependent production of the polyamine spermidine by DCs induces both IDO1 enzymatic and signaling activities, allowing the establishment of a tolerogenic phenotype in response to TGFβ (138). Interestingly, myeloid-derived suppressor cells also release polyamines that condition DCs to express IDO1 and, therefore, amplify the immune suppression exerted through joint modulation of amino acid catabolism in cancer (138).

Enhanced Trp and Arg catabolism causes amino acid depletion in the local microenvironment, which is sensed by T cells via the Ser/Thr kinase general control non-derepressible 2 kinase (GCN2) and results in limited protein synthesis and proliferative arrest (139–141). Intriguingly, GCN2 activation in response to amino acid scarcity improves antigen presentation by human moDCs *in vitro* in response to yellow fever vaccine YF-17D by enhancing autophagy (142). Indeed, human CD8+ T cell responses after YF-17D vaccination correlate with increased expression of GCN2 and autophagy-related genes, and mice deficient for GCN2 or autophagy related-proteins 5 or 7 in the CD11c compartment show impaired antiviral T cell responses (142). Hence, active Trp and Arg amino acid metabolism by DCs influences the microenvironment and T cell responses and is involved in immune suppression.

## Concluding Remarks

DCs are functionally defined by their ability to prime immunity and tolerance, but how their cellular metabolism (Figure 2) is affected by sensing of environmental cues and how this metabolic rewiring affects, in turn, DC function is an emerging fascinating field. The diversity of DCs (Tables 1, 2) and the fact that a great body of literature has been generated using DC-like cells from mouse bone marrow cultures with GM-CSF (12) limit our ability to predict what are the regulation and consequences of metabolic rearrangements in natural DCs *in vivo*.

Moreover, the use of inhibitors or genetic deletion of metabolic regulators to interrogate modulation of metabolic pathways is debated. Metabolic inhibitors have the advantage of immediate action on otherwise unaltered DCs and universal application on primary mouse and human DCs *ex vivo*. However, their applicability for DC-specific *in vivo* studies is limited, and they can have off-target effects, such as reported for C75, etomoxir, and 2-DG (16, 20, 58). On the other hand, genetic deletion of metabolic regulators in DCs using Cre-expressing mouse lines or other genetic approaches such as shRNA or CRISPR/Cas9 largely circumvents side effects and allows investigation of DCs with metabolic impairment *in vivo*. Nevertheless, genetic deficiency of important metabolic regulators can cause a deregulation of DC development (see the section Metabolic Control of Dendritic Cell Development) that challenges investigation of their functions, and unrelated compensating mechanisms that are difficult to control. While there is probably no consensus on the ideal strategy, studying consequences of manipulation of DC metabolism *in vivo*, rather than *in vitro*, may be of high relevance, as the microenvironment is crucial for cellular metabolism. Additionally, future studies of DC metabolism employing combined approaches of pharmacological inhibition and genetic deficiency will be most convincing.

Nevertheless, some patterns are starting to emerge showing that moDC and cDC1 generation is more dependent on functional mitochondrial metabolism and OXPHOS than cDC2s or pDCs (Tables 1, 2). Early induction of glycolysis characterizes and is required for immunogenic activation of cultured DCs and primary DC subsets, while long-term glycolytic reprogramming is finely regulated and may have suboptimal consequences (Figure 3). Indeed, important differences of metabolic/glycolytic adaptions of DCs early or late after stimulation are emerging, such as the different signaling pathways regulating early (49) and, likely, rather late glycolytic reprogramming (50, 56) or the time-dependent substrate use for glycolysis (46). Moreover, while weak and potent stimulants induce early glycolytic activity in GM-DCs, only strong activation achieves maintenance of increased glycolysis for 18 h or longer (56), further supporting the action of different mechanisms. Notably, metabolic flexibility for energy generation of long-term activated GM-DCs (3 days or more) seems to benefit their immunogenic functions (60, 64, 68) (Figure 3).

In contrast, tolerogenic DCs appear to generally rely more on OXPHOS than glycolysis, based on cultured human moDCs tolerized with specific stimuli. However, we only understand fragments of the cellular energy metabolism of tolerogenic DCs and the signaling pathways controlling their induction and maintenance of their functions.

Importantly, different DC subsets (Table 1) emerge to display pronounced variations in their adaption of mitochondrial energy metabolism upon immunogenic activation (Figure 4), reaching from strong induction of OXPHOS in pDCs, context-dependent alterations in cDCs, to a long-term reduction in cultured GM-DCs or human moDCs. Additionally, while enhanced glycolysis and FAS appear as general features of activated DCs, the further application of fatty acids as building blocks for the ER/Golgi or substrate for FAO also largely varies among DC subsets (Figure 4). Further efforts in primary DC subsets in different settings will likely contribute to a better understanding of context dependence and regulation of immunogenic and tolerogenic DC subset metabolism, as highlighted for lung inflammatory DCs (31).

Overall, integration of nutrient sensing and adequate adaption of mTOR/AMPK signaling (Figure 1) are crucial for metabolic adjustments by DCs. However, the complexity of metabolic reprogramming of DCs (upon stimulation) is highlighted by the fact that the signaling mechanisms involved in inducing glycolytic activity show context dependency and even contradictory effects with regard to regulating DC function. This controversy might be explained by differential routes of activation and additional functions and nutrient-dependent regulations of those important cellular signaling networks in DCs, apart from controlling glycolytic metabolism, that remain to be defined. For example, mTOR signaling is often linked with immunogenic DC activation due to increasing glycolytic and anabolic metabolism (41, 43, 59). However, tolerized moDCs also exhibit increased glycolysis compared to control moDCs in the steady state or after additional stimulation (88, 89), which was, indeed, also dependent on mTOR and reduced by rapamycin (91). Those observations indicate that the general association of a metabolic state, anabolic glycolysis vs. catabolic FAO/mitochondrial respiration (Figure 2), and concomitantly pathways controlling metabolic adaption to nutrients, mTOR vs. AMPK activation, cannot be generally ascribed to immunogenic vs. tolerogenic DCs.

Indeed, the influence of the particular immunogenic or tolerogenic context, ontogenic constraints of distinct DC subsets, and additional (environmental) factors on the balance of nutrient-sensing pathways and metabolic adaptions of DCs will have to be carefully assessed in the future.

## Author Contributions

SKW prepared tables and figures and conceptualized and wrote the manuscript. SCK conceptualized and wrote part of the manuscript. EP and IH-M helped conceptualize the manuscript and prepared the figures. DS contributed to funding acquisition and supervised, conceptualized, and wrote the manuscript. All authors declare no conflict of interest, contributed to manuscript revision, and read and approved the final version.

### Conflict of Interest Statement

The authors declare that the research was conducted in the absence of any commercial or financial relationships that could be construed as a potential conflict of interest. The handling editor declared a past co-authorship with one of the authors DS.
